# The AHL Quorum-Sensing System Negatively Regulates Growth and Autolysis in *Lysobacter brunescens*

**DOI:** 10.3389/fmicb.2019.02748

**Published:** 2019-12-03

**Authors:** Jun Ling, Lan Zhou, Guichun Wu, Yancun Zhao, Tianping Jiang, Fengquan Liu

**Affiliations:** ^1^Jiangsu Key Laboratory for Food Quality and Safety, State Key Laboratory Cultivation Base of Ministry of Science and Technology, Institute of Plant Protection, Jiangsu Academy of Agricultural Sciences, Nanjing, China; ^2^Academy of Agricultural Sciences of Yanbian, Longjing, China; ^3^School of the Environment and Safety Engineering, Jiangsu University, Zhenjiang, China

**Keywords:** acyl-homoserine lactone, AHL quorum sensing, regulation, growth rate, autolysis, *Lysobacter brunescens*

## Abstract

*Lysobacter* species are emerging as novel sources of antibiotics, but the regulation of their physiological metabolism is still poorly understood. In this work, we extracted AHL (acyl-homoserine lactone) autoinducers, identified the structures of AHLs and described the AHL quorum-sensing system in *Lysobacter brunescens* OH23. AHLs were isolated from the supernatant of *L. brunescens* OH23, and ESI-MS/MS (electrospray ionization mass spectrometry) analysis revealed biosynthesis of three different AHL chemical structures by *L. brunescens* OH23: *N*-(3-oxohexanoyl)- homoserine lactone (HSL), 3-OH-C_10_-HSL and C_8_-HSL. The growth rate of AHL quorum-sensing knockout mutants was dramatically increased compared to that of wildtype. Sucrose consumptions were also twice as high in AHL quorum-sensing knockout mutants than that in wildtype in early-log phase. Additionally, expression of key genes related to sucrose metabolism α-glucosidase was enhanced in AHL quorum-sensing knockout mutants, which indicated that AHL quorum sensing negatively regulates sucrose uptake and metabolism which further affects the growth rate of *L. brunescens*. Furthermore, autolysis was strongly induced in AHL quorum-sensing knockout mutants compared to wildtype, suggesting that AHL quorum sensing plays a negative regulatory role in cell autolysis. Moreover, compared to wildtype, XSAC (*Xanthomonas*-specific antibiotic compound) production was significantly increased in AHL knockout mutants in the early-log and late-log phases, and surface motility capabilities are also enhanced also in AHL knockout mutants; the normalized data of XSAC production and surface motility and expressions of key genes related to these two phenotypes reveal that growth rare and autolysis strongly affects XSAC biosynthesis and surface motility rather than AHL quorum-sensing system. Our results show that the AHL quorum-sensing system negatively regulates cell growth and autolysis, and further maintain nutrition homeostasis and population stability in *L. brunescens*.

## Introduction

*Lysobacter*, belonging to *Xanthomonadaceae*, is a type of gram-negative bacteria that inhibits the growth of a variety of plant pathogens by producing various active secondary metabolites (Folman et al., 2003). Lysobactin, originally isolated from *Lysobacter* sp. ATCC53042, inhibits a different pathogenic gram-positive and gram-negative bacteria (O’Sullivan et al., 1988). WAP-8294A, cyclic lipodepsipeptides, were isolated from *Lysobacter* sp. 8294 and inhibited clinical MRSA (methicillin-resistant *Staphylococcus aureus*) in mice better than did vancomycin (Kato et al., 1998; Zhang et al., 2011). In addition, Li et al. found that the highly thermostable antifungal factor HSAF (dihydromaltophilin) from *L. enzygenes* C3 inhibits various fungi (Yu et al., 2007).

Originally discovered in *X. campestris* pv. *campestris* (*Xcc*), the diffusible signal factor (DSF) quorum-sensing system is widely distributed among gram-negative bacteria, particularly *Xanthomonas campestris* and *Xanthomonas oryzae* (Barber et al., 1997; Wang et al., 2004). The DSF signaling molecule was identified as an unsaturated fatty acid with a chemical structure of *cis*-11-methyl-2-dodecenoic acid (Wang et al., 2004). Subsequently, it was discovered that *Xcc* can synthesize DSF molecules with at least three different structures, including DSF, *cis*-2-dodecenoic acid (BDSF) and [(2Z,5Z)-11-methyldodeca-2,5-dienoci acid] (CDSF) (He et al., 2010). Although the structures of DSF signaling molecules vary, their biosynthesis in *Xanthomonas* is dependent on the gene cluster *rpf* (regulation of pathogenicity factor) (He et al., 2006). Among the genes in this cluster, *rpfF* encodes an enoyl-CoA hydratase responsible for the biosynthesis of DSF signaling molecules (Almeida et al., 2012). In addition, the perception and conduction of DSF signals is dependent on the two-component system RpfC/RpfG in bacteria, namely, the histidine kinase (HK) RpfC and response regulator (RR) RpfG; RpfC is responsible for the perception of signaling molecules and RpfG for signal transduction (Slater et al., 2000).

The acyl-homoserine lactone (AHL)-mediated quorum-sensing system is also common in gram-negative bacteria, and the signaling molecules of the AHL-mediated quorum-sensing system are mainly synthesized by a class of genes homologous to *luxI* and *luxR*, which were the first quorum-sensing genes identified in *Vibrio fischeri* (Fuqua and Winans, 1994). The main function of the synthetic LuxI/LuxR quorum-sensing system in *V. fischeri* is regulation of luminescence according to the density of the population (Fuqua and Winans, 1994). In general, LuxI proteins are synthetases of AHLs and are responsible for the synthesis of related signaling molecules. LuxR family proteins are AHL molecular receptors, and the AHL autoinducers bind to LuxR proteins, which in turn regulate downstream gene expression and ultimately the relevant biological phenotype (Fuqua and Winans, 1994; Tsai and Winans, 2010). Typical LuxR-like receptor proteins require AHLs during protein folding or for evading hydrolysis by other hydrolases in the cell (Zhu and Winans, 1999, 2001). However, some special LuxR-like proteins do not depend on AHLs and, even in the presence of AHLs, inhibit the function of LuxR (Zhu and Winans, 1999, 2001). The AHL signaling molecules reported to date include N-(3-oxohexanoyl)-homoserine lactone (HSL) (*V. fischeri*), N-(3-oxooctanoyl)-HSL (*A. tumefaciens*), N-butanoyl-HSL (*Aeromonas hydrophila*), N-octanoyl-HSL (*Burkholderia cepacia*), N-(3-oxododecanoyl)-HSL (*Pseudomonas aeruginosa*), and N-hexanoyl-HSL (*Rhizobium leguminosarum*) (Miller and Bassler, 2001).

The quorum-sensing system is involved in cell-to-cell communication, regulates transcription factor activity and alters bacterial phenotypes, including biofilm, virulence, and symbiosis processes (Zheng et al., 2015; Papenfort and Bassler, 2016). During nodulation in *Mesorhizobium tianshanense*, the number of nodules per plant inoculated with AHL-QS mutants was significantly decreased compared to that inoculated with the wildtype strain, which indicated that the AHL quorum-sensing system was required during the symbiotic process between the bacterium and host plant *Glycyrrhiza* (licorice) (Cao et al., 2009). In *Burkholderia cepacian*, the AHL quorum-sensing system positively regulates swarming motility and biofilm formation, and complementation of the LuxR regulator *cepR* was able to restore swarming motility (Huber et al., 2001). In contrast, *Pseudomonas syringae* pv. *Actinidiae* (Psa) encodes three solo LuxR and negatively biosynthesize AHL autoinducers; Psa also senses the neighboring AHL-positive bacteria by responding to the AHLs signals, which might important for Psa fitness and virulence in the host kiwifruit (Cellini et al., 2019). In *Lysobacter enzymogenes*, overexpression of the solo LuxR type protein LesR led to a decrease in HSAF (Heat Stable Antifungal Factor) production, acceleration of cell aggregation and enhancement of melanin-like pigment production (Qian et al., 2014). In *Burkholderia glumae*, the AHLs quorum sensing system mutant grew more rapidly than wildtype strain and the glucose uptake was also increased in AHL QS mutant, which indicating that the AHL quorum sensing system mediated the homeostasis of nutrient metabolism of individual cells, especially under the nutrition limit condition (An et al., 2014).

In this study, we isolated AHL autoinducers and investigated the function of the AHL quorum-sensing system in *L. brunescens*. To this end, we disrupted the key genes *lbsI* and *lbsR* and assessed the growth, autolysis, motility and *Xanthomonas*-specific antibiotic compound (XSAC) production in *L. brunescens*. According to our results, the *lbsI* and *lbsR* mutants grew more rapidly than that of wildtype OH23 at the early-log phase, and the AHL quorum-sensing system altered the growth rate by modulating sucrose uptake. Moreover, autolysis in the *lbsI* and *lbsR* mutants occurred faster than that in wildtype, which indicated that the AHL quorum-sensing system negatively regulates autolysis in *L. brunescens*. In XSAC production and surface motility assays, the normalized data of XSAC production and surface motility showed no significant difference between wildtype OH23 and AHL knockout mutants. Moreover, expression of key genes related to XSAC production and surface motility did not differ, which indicated that the effects of growth and autolysis on these phenotypes are much greater than the direct regulation of AHL quorum sensing system. Taken together, AHL quorum sensing downregulates the growth rate and autolysis, indicating that AHL quorum sensing system is involved in regulation of nutrition consumption to maintain population stable and cooperative in *L. brunescens* OH23.

## Materials and Methods

### Bacterial Strains, Vectors and Culture Conditions

The bacterial strains and plasmids used in this study are listed in Table 1, and the primers used are listed in Table 2. *Escherichia coli* strains were grown on LB medium (Lysogeny Broth containing 10 g tryptone, 5 g yeast extract, and 10 g sodium chloride in 1 l of distilled water, pH 7.0-7.2) at 37°C (Sambrook and Russell, 2001). *L. brunescens* and its derivative mutants were grown on NB medium (Nutrient Broth containing 5 g tryptone, 1 g yeast extract, 3 g beef extract, and 10 g sucrose in 1 l of distilled water, pH 7.0–7.2) at 28°C (Atlas and Parks, 1997). The AHL detection strains were grown on AT medium [5 g glucose, 2 g (NH_4_)_2_SO_4_, 0.078 g MgSO_4_, 0.0076 g CaCl_2_, 0.005 g FeSO_4_⋅7H_2_O, 0.0022 g MnSO_4_⋅H_2_O, and 10.7 g KH_2_PO_4_ in 1 l of distilled water, pH 7.0–7.2] (Fuqua and Winans, 1994). All solid media contained 1.5% agar, and antibiotics were added at the following concentrations: rifamycin, 20 μg/ml; gentamicin, 8 μg/ml. Sucrose was added to a final concentration of 4% in media used for counterselection of the in-frame deletion strain and its derivatives. In-frame deletions of *lbsR* and *lbsI* were constructed by cloning the regions flanking *lbsR* and *lbsI* into the suicide vector pJQ200SK containing a *sacB* counterselectable marker. The resulting plasmids were introduced into *L. brunescens* by conjugation, and deletion mutants were selected for double homologous recombination events.

**TABLE 1:** Bacterial strains and plasmids.

**Strain/plasmid**	**Description**	**Source or reference**
***Lysobacter brunescens***		
OH23	Wildtype strain, strong specific activity against *Xanthomonas* species	Ling et al., 2019
OH23 Rif	Spontaneous Rif^R^ mutant of OH23, Rif^R^	Ling et al., 2019
OH23 (pBBR)	OH23 harboring the plasmid pBBR-*lbsR*, Rif^R^, Gm^R^	This study
Δ*lbsR*	*lbsR* gene in-frame deletion mutant	This study
Δ*lbsI*	*lbsI* gene in-frame deletion mutant	This study
Δ*lbsR* (*lbsR*)	Δ*lbsR* harboring the plasmid pBBR-*lbsR*, Rif^R^, Gm^R^	This study
Δ*lbsR* (pBBR)	Δ*lbsR* harboring the plasmid pBBR-MCS5, Rif^R^, Gm^R^	This study
Δ*lbsI* (*lbsI*)	Δ*lbsI* harboring the plasmid pBBR-*lbsI*, Rif^R^, Gm^R^	This study
Δ*lbsI* (pBBR)	Δ*lbsI* harboring the plasmid pBBR-MCS5, Rif^R^, Gm^R^	This study
***Xanthomonas***		
*Xanthomonas oryzae* pv. *oryzae*	Plant pathogen, causes bacterial leaf streak disease in rice	Song et al., 2015
RS105		
***E. coli***		
DH5αλpir	*supE44 Dlacu169 (f80 lacZDM15) hsdR17 recA1 endA1 gyrA96 thi-1 relA1* λpir	Kolter et al., 1978
S17-1λpir	Tp^R^ Sm^R^ *recA thi pro hsdR*^–^M^+^ *recA*:RP4-2-Tc:Mu Km:Tn7 λpir	Penfold and Pemberton, 1992
*E. coli* (*lbsI*)	*E. coli* DH5αλpir harboring the plasmid pBBR-*lbsI*, Gm^R^	This study
**Plasmids**		
pJQ200SK	Suicide cloning vector, Gm^R^	Quandt and Hynes, 1993
pBBR1-MCS5	Broad-host vector with the P*_*lac*_* promoter, Gm^R^	Kovach et al., 1995
pJQ-*lbsR*	pJQ200SK derivative carrying Δ*lbsR*:ΩGm, Gm^R^	This study
pJQ-*lbsI*	pJQ200SK derivative carrying Δ*lbsI*:ΩGm, Gm^R^	This study
pBBR-*lbsR*	pBBR1-MCS5 carrying *lbsR*, Gm^R^	This study
pBBR-*lbsI*	pBBR1-MCS5 carrying *lbsI*, Gm^R^	This study

**TABLE 2:** Primers used in this study.

**Primer name**	**Sequence (5′→ 3′)**	**Usage**
*lbsR*-1	TCCTGCAGCCCGGGGGATCCCCACGTGCAGGCCGAGGTGG	*lbsR* deletion
*lbsR*-2	GGGTCATGTGAGCGCCTGAGCCCGCGGGCGCGATC	
*lbsR*-3	GCGCTCACATGACCCCTGTTCCCGATTC	
*lbsR*-4	GCGGCAGCGGCCGCTCTAGACACGGCGACGAAATCGACGC	
*lbsI*-1	TCCTGCAGCCCGGGGGATCCCGATGTTCCAGAGC	*lbsI* deletion
*lbsI*-2	TGACCCGTCCCGCGCAGCCTGA	
*lbsI*-3	GCGCGGGACGGGTCATGGTGTCTCC	
*lbsI*-4	GCGGCAGCGGCCGCTCTAGAAAGACGCCCGCGCAGT	
*lbsR*-cp-1	CGACGGTATCGATAAGCTTCATGGGTGGCAGGACGCTGAT	*lbsR* complementation
*lbsR*-cp-2	CCACCGCGGTGGCGGCCGCTCTAGATCAGTTGCGGAAGGTGGAGA	
*lbsI*-cp-1	AGGTCGACGGTATCGATAAGCTTCATGACCCGTATTGCCATCG	*lbsI* complementation
*lbsI*-cp-2	GCGGTGGCGGCCGCTCTAGATCAGGCTGCGCGGGCGATGT	
RT-*peg.977*-1	GACAGCTGGTTGCCGGAATG	*peg.977* expression detection
RT-*peg.977*-2	CGGCGACGGCGAATTCGATT	
RT-*pilA*_1_-F	AAGCCGAACGTCCAGATATC	*pilA*_1_ expression detection
RT-*pilA*_1_-R	GGCTGGAATTCGAGGAATAC	
RT-*peg.1602*-1	GATCGACCATGCCTGGTTCC	*peg.1602* expression detection
RT-*peg.1602*-2	TGCGGGTTGTGGAAGTTCAG	
RT-*peg.2863*-1	TGCTCGCTGAGGAACCCATC	*peg.2863* expression detection
RT-*peg.2863*-2	ATTGCGTGCAGACGATCTAC	
RT-*recA*-1	GTCACCGAAATCCTCTATGG	*recA* expression detection
RT-*recA*-2	GGGTTGTCCTTCATGTACTG	

### AHL Bioassays and Identification

To detect whether *L. brunescens* biosynthesizes AHL autoinducers, a highly efficient detection strain, *A. tumefaciens* KYC55 (pJZ372) (pJZ384) (pJZ410), was used to examine the biosynthetic ability of OH23. The autoinducer synthase gene *traI* in the highly efficient detection strain was disrupted, and a plasmid (P*_*T*__7_*-*traR*) capable of overexpressing the *A. tumefaciens* TraR protein and a reporter plasmid containing the P*_*traI*_*-*lacZ* promoter were introduced. The combination of TraR and AHL autoinducers convert it to an active form that binds to the promoter sequence of *traI*, initiating its transcription and expression of *lacZ*. Therefore, overexpression of TraR increases its binding to the autoinducer, enhances expression of the reporter *lacZ* gene and increases the sensitivity of the detection strain to the autoinducer (Zhu et al., 2003).

*L. brunescens* and AHL mutants were grown in NB liquid medium at 28°C at 180 rpm; the cultures were collected at the indicated time points and centrifuged at 12,000 rpm for 10 minutes, and the supernatant was extracted with the same volume of ethyl acetate, as previously described (Ling et al., 2019). Two liters of the organic phase of every treatment was evaporated at 47°C, and the crude extract was dissolved in 2 ml dimethyl sulfoxide (DMSO). Two microliters of the crude extract and approximately 10^7^ AHL bioassay strain cells per ml were added to AT medium and incubated at 28°C at 180 rpm for 12 h followed by assays for β-galactosidase specific activity (Miller, 1972; Fuqua and Winans, 1994; Zhu and Mekalanos, 2003). A thin-layer chromatography (TLC) assay was used to analyze the AHLs biosynthesized by *L. brunescens* strains, as previously described (Zheng et al., 2015), using a sensitive AHL indicator strain. Briefly, concentrated crude extracts were applied to C_18_ reversed-phase TLC plates (Whatman) and incubated at 28°C for 12 h.

For the identification of AHLs, the supernatant was extracted with the same volume of chromatographically pure dichloromethane, and AHLs were identified by ESI-MS/MS. The structures of AHLs were calculated by the following formulas: for C_*n ×2 + 4*_-HSL, *n* = (*m*/*z*-1-171)/28; for 3-hydroxyl-C_*n×2+4*_-HSL, *n* = (*m*/*z*-1-187)/28; for 3-oxo-C_*n×2+4*_-HSL, *n* = (*m*/*z*-1-185)/28 (Yang et al., 2009).

### Growth Measurements

*L. brunescens* OH23 and the AHL knockout mutants were cultured in NB medium at 28°C with shaking at 180 rpm until the OD_600_ was approximately 1.0 [which corresponds to approximately 10^9^ CFU/ml (Colony Forming Units/ml), about 18 to 22 h]. One milliliter of culture for each strain was transferred to 50 ml of new liquid NB medium, and the cultures were incubated at 28°C with shaking at 180 rpm. To measure growth, the OD_600_ value was determined every 12 h for each culture using a BioPhotometer Plus (Eppendorf, Germany) until each culture reached the stationary phase. Three replicates were performed for each treatment, and the experiment was repeated three times.

### Sucrose Consumption Assays

One milliliter (OD_600_ was approximately 1.0, about 18 to 22 h) of culture for test strain was transferred to 50 ml of new liquid NB medium and incubated at 28°C with shaking at 180 rpm; samples were collected every 12 h. The cells were removed by centrifugation, and the supernatants were used to analyze the concentrations of sucrose by using a sucrose detection kit (Solarbio, Beijing Solarbio Science & Technology Co., Ltd.).

### Autolysis Assays

Autolysis assays were performed as described by Mani et al. with modifications (Chitnis et al., 1990; Mani et al., 1993). Bacteria were grown in NB liquid medium at 28°C with shaking at 180 rpm to an OD_600_ of approximately 0.7 (about 16 to 18 h). The cells were washed twice with PBS, resuspended in the same volume of PBS and incubated at 28°C with shaking at 180 rpm. Optical density was measured at the indicated time points. The results were normalized to an OD_600_ at time zero, and percent lysis was calculated using the formula% autolysis = [OD_600_ (*t*_0_)-OD_600_ (*t*)]/OD_600_ (*t*_0_).

### Effect of the AHL Quorum-Sensing System on XSAC Production

The ability of the wildtype and AHL mutants to produce XSAC was measured by an anti-*Xanthomonas* activity assay (diameter of inhibition zone), which was described previously (Ling et al., 2019). Briefly, the pathogenic strain *X. oryzae* pv. *oryzae* RS105 was incubated in NB liquid medium at 28°C with shaking at 180 rpm until the culture OD_600_ was approximately 1.0; *L. brunescens* OH23, Δ*lbsR* and Δ*lbsI* mutants were incubated in NB liquid medium at 28°C with shaking at 180 rpm until the indicated time points. For *X. oryzae* pv. *oryzae* RS105, 100 ml of liquefied NB solid medium was incubated at 45°C for 30 min, mixed with 10^8^ cells, and then poured into plates for the anti-*Xanthomonas* activity assay. The cultures of *L. brunescens* OH23, Δ*lbsR* and Δ*lbsI* were centrifuged, and the supernatants were separately incubated at 85°C for 30 min; 30 μl of the treated supernatant from the cultures at the indicated time points was added to the hole in the selection plates. All plates were cultured at 28°C, and the zones of inhibition were photographed and compared after 2 days. XSAC production was evaluated, as represented by the diameter of the inhibition zone. Three replicates were performed for each treatment, and the experiment was repeated three times.

### Observation of Surface Motility

The surface motility assay of *L. brunescens* wildtype OH23, Δ*lbsR* and Δ*lbsI* was performed as previously described (Ling et al., 2019). Briefly, NB semi-solid medium containing 0.3% agar was used, and 2.5 μl of *L. brunescens* wildtype OH23, Δ*lbsR* or Δ*lbsI* (10^9^ CFU/ml, OD_600_ was approximately 1.0 for all strains, about 18 to 22 h) was spotted onto the surface. The plates were incubated at 28°C for 4 days, and the surface motility of each strain was photographed, measured, and compared. Three replicates were performed for each treatment, and the experiment was repeated three times.

### RNA Extraction, Reverse Transcription PCR, and Real-Time PCR

*L. brunescens* OH23, Δ*lbsR* and Δ*lbsI* mutants were each grown in 5 ml NB liquid medium until the OD_600_ was approximately 1.0 (about 18 to 22 h) or until to the indicated time points. Three milliliters of cells were transferred to a sterile centrifuge tube and centrifuged for 3 min at 12,000 rpm. RNA was extracted using TRIzol solution (TaKaRa Biocompany) following the manufacturer’s instructions and 250 ng RNA of every sample was used for further experiments. For DNA removal and reverse transcription PCR, HiScript III RT SuperMix for qPCR (+ gDNA wiper) (Vazyme Biotech Co., Ltd.) was used in this study. The real-time PCR assay was carried out as previously described (Ling et al., 2019). A QuantStudio 6 Flex Real-Time PCR System (Thermo Fisher Scientific) and HiScript II One Step qRT-PCR SYBR Green Kit (Vazyme Biotech Co., Ltd.) were used to detect gene expression. Gene expression was calculated by the 2^−ΔΔ*CT*^ method, and *recA* cDNA was used as an internal control in all reactions. The primers used for real-time PCR are listed in Table 2. Three replicates were performed for each treatment, and the experiment was repeated three times.

### Data Analysis

Statistical analyses were calculated using SPSS (Statistical Package, Version 21.0). The variables were subjected to Student’s *t* test and tested for significance at *P* < 0.05 (^∗^), *P* < 0.01 (^∗∗^), *P* < 0.001 (^∗∗∗^), and *P* < 0.0001 (^****^).

## Results and Discussion

### Identification of AHL Molecules in *L. brunescens*

**FIGURE 1:**
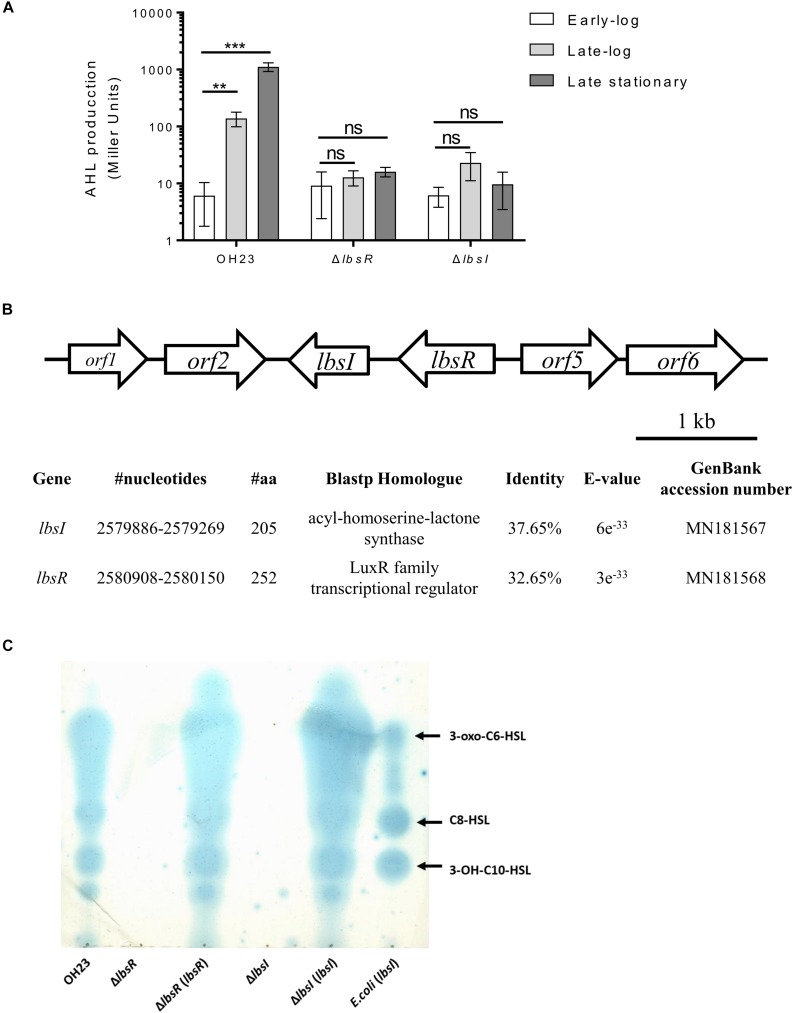
The location of the AHL *lbsI/lbsR* quorum-sensing system in *L. brunescens* OH23 and the AHL structures and AHLs of *L. brunescens* OH23 and its mutants. **(A)** AHL production in *L. brunescens* and its QS mutants in different growth phases. Statistical analyses of AHL production were performed using Student’s *t*-test compared with wildtype OH23 at the early-log phase. ^∗∗^*P* < 0.01; ^∗∗∗^*P* < 0.001; and ns (ns: no significance), *P* > 0.05. **(B)** Physical map of the LbsR/LbsI quorum sensing system gene cluster in *L. brunescens*. The identity results were represented by BLASTp comparison between the proteins encoded by *lbsI* and *lbsR* of *L. brunescens* and *P. aeruginosa* (taxid: 287). The protein similarity was compared using BioEdit 7.0.9.0. **(C)** TLC analysis of AHL contents. The supernatants of wildtype OH23, Δ*lbsI*, Δ*lbsR*, Δ*lbsI* (*lbsI*), Δ*lbsR* (*lbsR*) and *E. coli* (*lbsI*) were extracted and loaded onto a C_18_ reverse-phase TLC plate, followed by an overlay of the agar medium seeded with JZA1 bioassay strains. Each lane was loaded with 2 μl of concentrated extract (Miller Unit ≈ 500).

Wildtype OH23 was grown in 10 l NB liquid medium until the OD_600_ was approximately 2.0, which corresponds to approximately 2 × 10^9^ CFU/ml. Extraction of the crude AHLs from the supernatant was performed using the same method previously described (Holden et al., 1999), and the extracted AHL signaling activities in *L. brunescens* OH23 were detected by an *Agrobacterium*-based AHL bioassay (Zhu et al., 2003). The AHL autoinducers from the wildtype strain OH23 had higher induction capabilities, and *lacZ* expression was dramatically induced in *Agrobacterium*. Furthermore, we detected autoinducer activity in different cell growth stages. As shown in Figure 1A, autoinducer production by wildtype was at a low level in the early-log phase; however, as the density of the cells increased, the concentration of autoinducer also increased, and AHL induction activities reached the highest value in the late-log phase. In *Mesorhizobium tianshanense*, the concentration of AHL signaling molecules increased with the growth of the strain and reached the highest concentration during the stationary phase (Zheng et al., 2006). The AHL autoinducers could be detected in *L. brunescens* supernatants, and AHL autoinducers show lower activity at low cell concentration and higher activity at high cell concentration which is a typical phenomenon of cell-density-dependent AHL quorum sensing system.

To confirm the *L. brunescens* AHL structures, the culture supernatant of the wildtype OH23 strain was extracted with dichloromethane, and concentrated AHLs were subjected to ESI-MS/MS analysis. Analysis of the mass spectrum revealed that the wildtype strain of *L. brunescens* mainly biosynthesized three types of AHL molecules, 3-OH-C_10_-HSL (MW271), C_8_-HSL (MW 227) and 3-oxo-C_6_-HSL (MW 213), with distinct peak times (Supplementary Figure S1).

### Identification of the AHL Quorum-Sensing System in *L. brunescens*

As mentioned above, AHL autoinducer signals have been reported in different bacteria, and three AHL autoinducers were isolated from *L. brunescens*. To identify genes related to AHL biosynthesis, random transposon insertional mutagenesis was used to screen AHL-deficient mutants (Supplementary Table S1). All transconjugants were grown in NB liquid medium until the OD_600_ was approximately 1.0, and supernatants were concentrated and used for liquid AHL bioassays. From approximately 3000 random transconjugants, 2 mutants exhibited deficiency in inducing the β-galactosidase activity of the *A. tumefaciens* indicator strain (Supplementary Figure S2A), indicating that the transposon-inserted gene might be responsible for AHL biosynthesis. The transposon flanking region was sequenced to identify genes potentially related to the AHL biosynthesis pathway (Supplementary Figures S2B,C). Based on BLAST searches of the transposon flanking region^1^, we found that the AHL-related proteins from *L. brunescens* have homology to those of *P. aeruginosa* (taxid: 287), with levels of protein identity of 32.62% (LbsR) and 37.65% (LbsI). LbsR is a LuxR family transcriptional regulator involved in AHL biosynthesis modulation, and LbsI is a GNAT family N-acetyltransferase responsible for the biosynthesis of AHLs (Figure 1B).

In *Mesorhizobium tianshanense* and *Pseudomonas syringae*, the disruption of LuxR-type regulator and LuxI-type synthase leaded to the AHL biosynthesis capabilities abolished (Quinones et al., 2004; Zheng et al., 2006). Meanwhile, the expression of MrtI requires the intact MrtR and AHL signals in *M. tianshanense* (Zheng et al., 2006). To demonstrate the role of the LbsR/LbsI system in the biosynthesis of AHL signaling molecules in *L. brunescens*, in-frame knockout experiments were performed on *lbsI* and *lbsR*, and the concentrated supernatant AHL activity of the mutants was evaluated. As shown in Figure 1A, mutation of *lbsI* and *lbsR* abolished the ability to produce AHLs during the entire bacterial life cycle, which suggested that *lbsI* and *lbsR* are essential for AHL biosynthesis in *L. brunescens*. Further confirmation by a TLC assay showed that the *lbsI* and *lbsR* mutants completely lacked the ability to produce any AHL molecules (Figure 1C). Two possible reasons that the *lbsI* and *lbsR* mutants abolished AHL biosynthesis capabilities under testing conditions. One is that the LbsI is responsible for the biosynthesis of AHL, which means that *lbsI* is indispensable in AHL biosynthesis. The second possible reason is that the regulation of LbsR protein is required in the synthesis of AHL signaling molecules, just like MrtR-MrtI regulation system in *M. tianshanense* (Zheng et al., 2006). *lbsI* and *lbsR* gene overexpression plasmids were transferred into the *lbsI* and *lbsR* knockout mutants, respectively, by conjugation. As shown in Figure 1C, the complemented strains Δ*lbsR* (*lbsR*) and Δ*lbsI* (*lbsI*) displayed strong activities in inducing *A. tumefaciens* indicator strain β-galactosidase expression compared to that treated by concentrated supernatants from Δ*lbsR* and Δ*lbsI*. The AHL TLC bioassay results indicated restoration of the biosynthesis activity of the AHL autoinducer in the deletion mutants.

To further understand the function of the AHL synthase gene *lbsI*, the gene was overexpressed under the P*_*lac*_* promoter in the broad-host vector pBBR1-MCS5 and electrotransformed into *E. coli* DH5α to obtain *E. coli* (*lbsI*). The culture supernatants were concentrated and tested for AHL activities with the *A. tumefaciens* indicator strain. As illustrated in Figure 1C, TLC assays revealed three blue halos for the culture supernatant of *E. coli* (*lbsI*), which indicated that *lbsI* is responsible for the synthesis of three AHL molecules in *E. coli* (*lbsI*), and this result was consistent with the ESI-MS/MS results.

### The AHL Quorum-Sensing System Modulates Sucrose Metabolism to Regulate Cell Growth in *L. brunescens*

**FIGURE 2:**
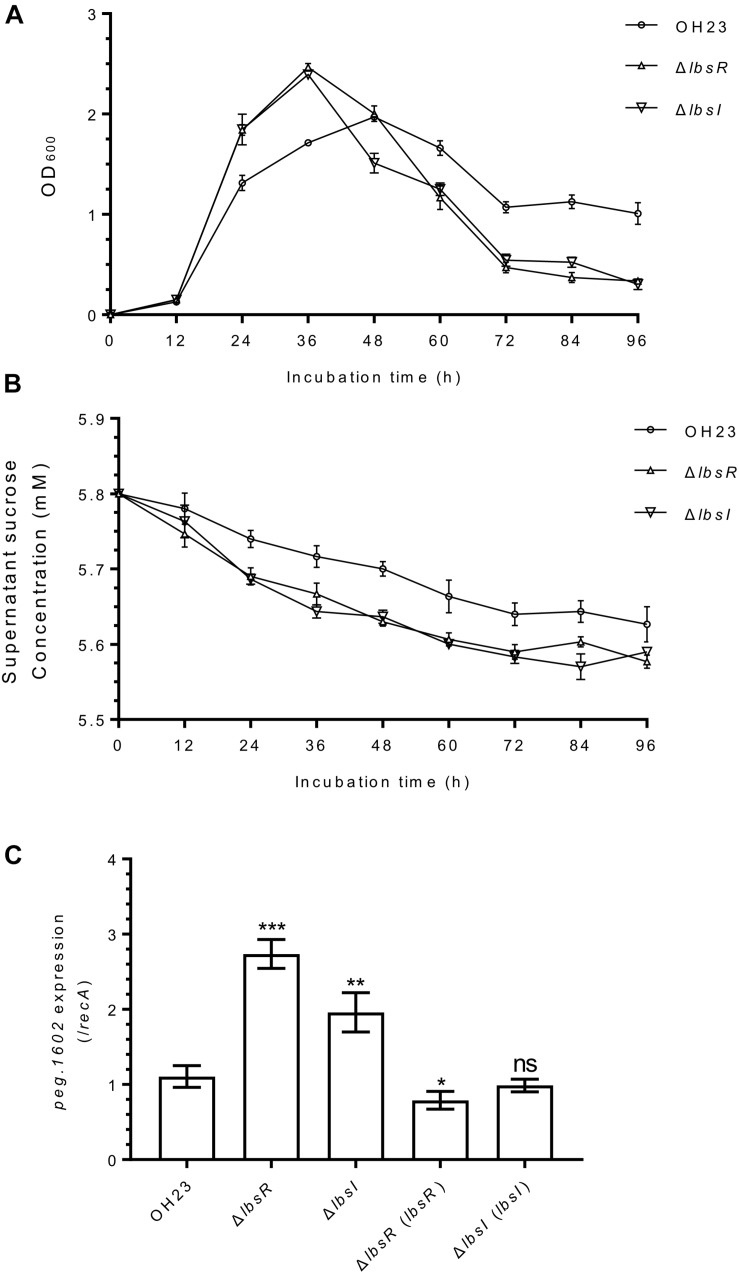
The growth and sucrose consumption-related gene expression of *L. brunescens* OH23 and its AHL mutants. **(A)** The growth of wildtype OH23 and its AHL mutants. Wildtype OH23 and AHL mutants (OD_600_ of approximately 1.0) were grown in NA liquid medium at 28°C, and the OD_600_ was measured at the time points indicated. The data are the combination of three individual experiments. **(B)** Sucrose consumptions of wildtype OH23 and its AHL mutants. The samples from growth measurement assay were centrifuged and the supernatants were used for sucrose concentration detection by using sucrose detection kit. **(C)** α-glucosidase gene *peg.1106* expression in wildtype OH23 and AHL mutants. The growth of mutants was compared with that of strain OH23 at the same time points. ^∗^*P* < 0.05; ^∗∗^*P* < 0.01; ^∗∗∗^*P* < 0.001; and ns (ns: no significance), *P* > 0.05 (Student’s *t*-test).

To investigate whether AHL quorum sensing regulates the growth rate in *L. brunescens*, we measured the growth of wildtype OH23, Δ*lbsR*, Δ*lbsI*, and the corresponding complementation strains in NB medium (Figure 2A, Supplementary Figure S4A, and Supplementary Table S1). The Δ*lbsR* and Δ*lbsI* knockout mutants grew faster in the early-log and late-log phase than that in wild-type OH23; the cell density of the mutant strain significantly decreased during the stationary phase compared to that of the wild type strain (Figure 2A). In the early exponential growth phase, the growth rates of Δ*lbsR* (pBBR) and Δ*lbsI* (pBBR) increased faster than that of OH23 (pBBR), which is consistent with the results observed for the Δ*lbsR* and Δ*lbsI* mutants (Supplementary Figure S4A). Moreover, no significant difference in growth rate was found between the complementation strains Δ*lbsR* (*lbsR*) and Δ*lbsI* (*lbsI*) and OH23 (pBBR) (Supplementary Figure S4A) and Δ*lbsI* mutant could show the same growth rate with wildtype OH23 in the condition of adding 2 μM C8-HSL (Supplementary Figure S4B).

AHL quorum-sensing knockout mutants of *Burkholderia glumae* grow more rapidly than do wildtype at the early exponential stage, and the AHL quorum-sensing system modulates the growth rate by invoking a phosphoenolpyruvate-dependent sugar phosphotransferase system (PTS) to affect the glucose uptake rate (An et al., 2014). In *X. axonopodis* pv. *glycines*, a unique sucrose hydrolase was identified that is responsible for sucrose metabolism to glucose and fructose (Kim et al., 2004). To determine whether the AHL-quorum sensing system uses sucrose metabolism to regulate the growth rate, we first detected the sucrose consumption in wildtype OH23 and AHL knockout mutants. As the strain grows, the sucrose content in the supernatant gradually decreases (Figure 2B). At 48 h, the sucrose consumption in the AHL knockout mutant strain was approximately twice than that of the wildtype OH23. In the later stage of growth, as the growth of AHL knockout mutants gradually slowed down in AHL knockout mutants, and the difference in sucrose consumption between AHL knockout mutants and wildtype OH23 gradually decreased. We also found a α-glucosidase responsible for sucrose metabolism (Supplementary Figure S3), and homology analysis revealed that *peg.1602* (GenBank number: MN557391), encoding 540 amino acids, is homologous to α-glucosidase from *Lysobacter* sp. TY2-98 (85%), with an *E*-value of 0.0. The enzyme encoded by *peg.1602* showed strong activity in digesting sucrose to glucose and fructose (Supplementary Figure S3B and Supplementary Table S1). Therefore, we detected expression of *peg.1602* to evaluate the sucrose metabolism activity in *L. brunescens*. Expression of *peg.1602* in OH23, Δ*lbsR*, Δ*lbsI* and complementation strains was detected using real-time PCR. As shown in Figure 2C and Supplementary Figure S4C, *peg.1602* expression was significantly higher in Δ*lbsR* and Δ*lbsI* than in wildtype OH23, whereas *peg.1602* expression did not differ significantly between wildtype OH23, the complementation strains and chemical complementation of Δ*lbsI*, which indicated that the AHL quorum-sensing system is involved in the regulation of *peg.1602* expression. The classical AHL QS system positively regulates the growth in *M. tianshanense* (Cao et al., 2009). Meanwhile, some AHL QS system also negatively regulates the growth in *B. glumae* and the AHL QS system might involve in the regulation of consumption of nutrition (An et al., 2014). Based on AHL quorum sensing system, the cells could sense other bacteria by the AHLs from intercellular and control the population size and nutrition consumption. Taken together, the AHL quorum-sensing system regulates the growth rate and restricts the sucrose consumption in *L. brunescens*, indicating that AHL quorum sensing system plays important roles in coordinating nutrition distribution in the population group to achieve stability and cooperation of the population, especially in the nutritional limit conditions.

### The AHL Quorum-Sensing System Negatively Regulates Autolysis in *L. brunescens*

**FIGURE 3:**
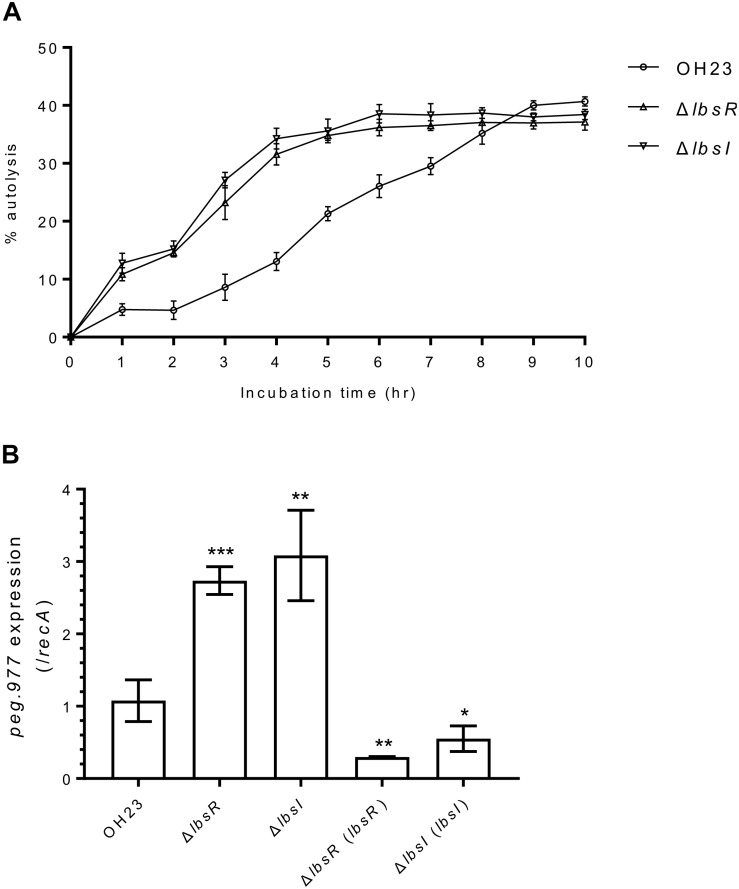
The autolysis and peptidoglycan hydrolase-related gene expression of *L. brunescens* OH23 and its AHL mutants. **(A)** Autolysis of wildtype OH23 and AHL mutant whole cells in PBS buffer (pH 7.2). 5 × 10^8^ cells of wildtype OH23 and its AHL mutants were washed twice with PBS (pH 7.2) and resuspended in equal volume PBS. The cultures were incubated at 28°C with shaking at 180 rpm. OD_600_ was measured at the indicated time points. The data are the combination of three individual experiments. **(B)** Peptidoglycan hydrolase *peg.977* expression in wildtype OH23 and AHL mutants. The data are the combination of three individual experiments. Statistical analyses of *peg.977* expression were performed using Student’s *t*-test compared with wildtype OH23. ^∗^*P* < 0.05; ^∗∗^*P* < 0.01; and ^∗∗∗^*P* < 0.001 (Student’s *t*-test).

Because the OD_600_ value of the AHL quorum-sensing knockout mutants was significantly lower than that of wildtype OH23 in the late stationary phase (Figure 3A), we speculated that AHL quorum sensing may be involved in the autolysis process in *L. brunescens*. During autolysis, peptidoglycan hydrolase digests the cell wall, which leads to a decrease in turbidity (OD_600_); thus, a turbidimetric assay can be used to calculate the degree of autolysis. As presented in Figure 3A, autolysis processes began at 1 h in wildtype and the Δ*lbsR* and Δ*lbsI* mutants, with percentages of 4.73, 10.84, and 12.79%, respectively. In addition, autolysis percentages in the Δ*lbsR* and Δ*lbsI* mutants reached over 30% after 4 h of incubation, whereas the wildtype strain required over 7 h to achieve 30% autolysis. In *S. aureus*, upregulation of the peptidoglycan hydrolase gene *lytM* further promotes autolysis (Michel et al., 2006). Homology analysis revealed that *peg.977* (GenBank number: MN557390), encoding 367 amino acids, is homologous to peptidoglycan hydrolase gene *lytM* from *S. aureus* (55%), with an *E*-value of 2 × e^–014^. To further confirm that AHL quorum sensing regulates autolysis in *L. brunescens*, we examined *peg.977* expression in wildtype OH23, the Δ*lbsR*, Δ*lbsI* mutants and chemical complementation of Δ*lbsI* strain. The results showed the *peg.977* expression increases ∼3-fold in the Δ*lbsR* and Δ*lbsI* mutants compared to wildtype OH23; the *peg.977* expression is downregulated in Δ*lbsR* (*lbsR*) and Δ*lbsI* (*lbsI*); the *peg.977* expression shows no significant difference between wildtype OH23 and chemical complementation of Δ*lbsI* strain (Figure 3B and Supplementary Figure S5). Taken together, AHL quorum sensing negatively regulates autolysis to control and maintain the population in *L. brunescens*.

### Growth Rate Accelerates Antibiotic XSAC Biosynthesis and Surface Motility Progress in *L. brunescens*

**FIGURE 4:**
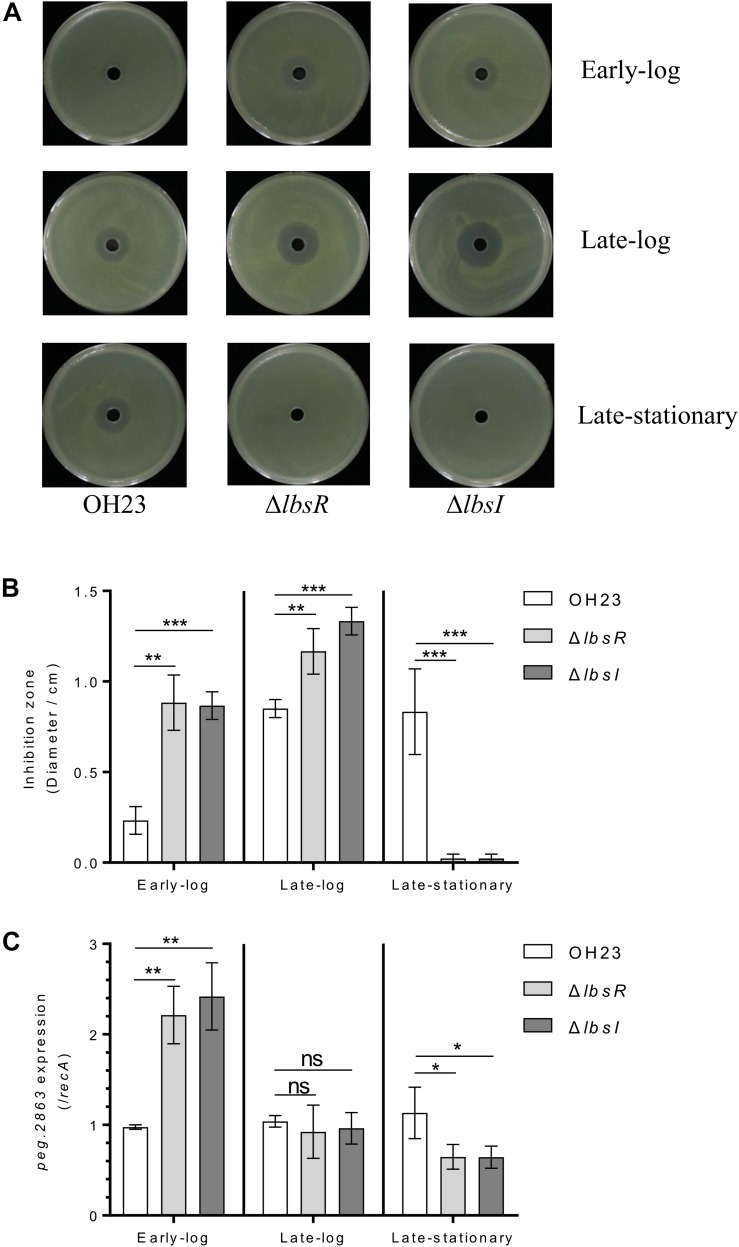
XSAC production in *L. brunescens* OH23 and its AHL mutants. **(A)** Cultures of *L. brunescens* OH23 or AHL mutants were tested for antimicrobial activity against *X. oryzae* pv. *oryzae* RS105 at the indicated time points. 10^5^ CFU cells of *X. oryzae* pv. *oryzae* RS105 were spread onto the surface of each NB plate, and 30 μl of test supernatants of *L. brunescens* and AHL mutants (samples collected from indicated time points) was added into the central holes of NB plates. The plates were incubated at 28°C for 4 days and the size of inhibition zones were using to calculate for the antimicrobial activity. **(B)** Analysis of the images of *X. oryzae* pv. *oryzae* RS105 growth inhibition zones shown in **A**. **(C)** *peg.2863* (key gene involved in XSAC biosynthesis) expression in wildtype OH23, Δ*lbsI* and Δ*lbsR*. ^∗^*P* < 0.05; ^∗∗^*P* < 0.01; ^∗∗∗^*P* < 0.001; and ns, *P* > 0.05 (Student’s *t*-test).

In a previous study, the AHL-type quorum-sensing system was found to affect the growth rate, swimming motility and infection virulence of *Acidovorax avenae* (Fan et al., 2011). Moreover, the AHL-type quorum-sensing LuxR family-type regulator is required for rice virulence in *Xanthomonas oryzae* pv. *oryzae* and negatively regulates heat-stable antifungal factor (HSAF) biosynthesis in *Lysobacter enzymogenes* (Ferluga et al., 2007; Qian et al., 2014). To investigate whether the AHL quorum-sensing system modulates XSAC biosynthesis in *L. brunescens*, we detected the anti-*Xanthomonas* abilities of wildtype OH23, Δ*lbsR* and Δ*lbsI* to evaluate the production of XSAC biosynthesis. As depicted in Figures 4A,B, anti-*Xanthomonas* abilities increased with the growth of OH23, and the diameters of the inhibition zone were 0.23 ± 0.08 cm, 0.85 ± 0.05 cm, and 0.83 ± 0.24 cm from the early-log phase to the late log phase to the stationary phase, respectively. Compared to wildtype OH23, the diameters of the inhibition zones of Δ*lbsR* and Δ*lbsI* increased 278.57 and 271.43% in the early-log phase and 37.25 and 56.86% in the late-log phase, respectively. In the late stationary phase, XSAC production was dramatically decreased in the Δ*lbsR* and Δ*lbsI* mutants, and the diameters of the inhibition zones were 0.07 ± 0.02 cm and 0.05 ± 0.01 cm, respectively.

To further confirm whether the AHL-type quorum-sensing system controls XSAC biosynthesis, we collected wildtype OH23, Δ*lbsR* and Δ*lbsI* cells at different cell growth phased and extracted RNA to detect expression of *peg.2863* (a key gene related to the biosynthesis of XSAC) (Ling et al., 2019). In the early-log phase, the expression of *peg.2863* in AHL knockout mutants were significantly enhanced comparted to wildtype Oh23; in the mid-log phase, expression of *peg.2863* showed no significant difference between wildtype OH23 and Δ*lbsR* or Δ*lbsI*.; in the late-stationary, the *peg.2863* expression was downregulated in AHL knockout mutants (Figure 4C). Combining the different growth rates presented in Figure 2A, the diameter of inhibition zone was normalized by the OD_600_, and the inhibition activities in late-log phase were shown no significant difference and between wildtype and AHL knockout mutants (Supplementary Figure S6A), which indicating that cell densities decrease dramatically in the AHL mutants due to autolysis in the late stationary phase and cause the AHL mutants to completely lack activity against *Xanthomonas oryzae* pv. *oryzae* RS105. In *L. enzymogenes*, the yield of HSAF accumulates with growth and reaches a maximum concentration at the stationary phase (Tang et al., 2018). In addition, the biosynthesis of HSAF is regulated by the DSF quorum sensing system in *L. enzymogenes*, and the loss of the key DSF quorum sensing system genes leads to a significant decrease in HSAF production (Han et al., 2015). Similarly, in *L. brunescens*, the capability of XSAC biosynthesis is completed lost in DSF quorum sensing knockout mutants (Ling et al., 2019). The inhibition zones of AHL knockout mutants was significantly bigger than that in wildtype OH23 in early-log and late-log phase; the differences of antagonistic capabilities between AHL knockout mutants and wildtype OH23 are not significant when the yield of all XSAC was normalized by OD_600_ value. The AHL quorum-sensing system is involved in the growth and autolysis of *L. brunescens*, and the growth rate and autolysis negatively affect XSAC biosynthesis. Taken together, the different XSAC production capacities between wildtype OH23 and the AHL mutants might be due to the different growth rates in the early-log phase and late-log phase.

**FIGURE 5:**
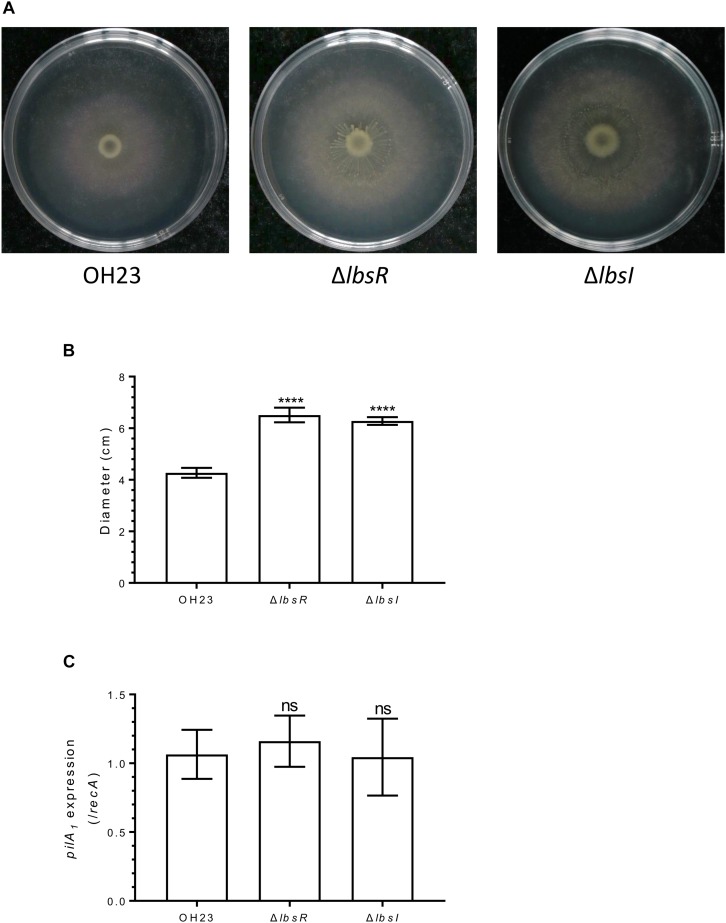
Surface motility in *L. brunescens* OH23 and its AHL mutants. **(A)** Surface motility of wildtype OH23 and AHL mutants. Each strain was grown in liquid NB medium until the culture reached an OD_600_ of approximately 1.0, after which 3 μl was inoculated onto the surface of 0.3% Agra NB plates. The plates were inoculated at 28°C for 4 days. **(B)** Analysis of the images of surface motility shown in **A**. **(C)** *pilA*_1_ expression in wildtype OH23, Δ*lbsI*, and Δ*lbsR*. ^****^P < 0.0001; and ns, *P* > 0.05 (Student’s *t*-test).

In *Pseudomonas syringae*, the AHL-mediated quorum-sensing system negatively regulates swarming motility (Quinones et al., 2005). To investigate whether the AHL-type quorum-sensing system participates in surface motility in *L. brunescens*, we tested the surface motility of wildtype OH23, Δ*lbsR* and Δ*lbsI* on NB semi-solid (0.3% agar) motility medium plates for 4 days at 28°Ñ. As illustrated in Figures 5A,B, the surface motility diameter of wildtype OH23 was 4.27 ± 0.17 cm over four days. However, the average surface motility diameters of Δ*lbsR* and Δ*lbsI* reached 6.51 ± 0.25 cm and 6.28 ± 0.13 cm, respectively. In order to eliminate side-effects of growth rates, the diameter of surface motility was normalized by the OD_600_. And the diameters of surface motility were shown no significant difference and between wildtype and AHL knockout mutants (Supplementary Figure S6B). Moreover, *pilA*_1_ gene expression showed no significant difference between wildtype OH23 and the AHL-type QS mutants, indicating that surface motility was indirectly regulated by the AHL-type quorum-sensing system and might be affected by the growth rate in *L. brunescens* (Figrue 5C). In Psa, when, the motility of the bacteria and the expression of some genes related to motility are significantly enhanced while adding 1 μM of AHL signaling molecules. In addition, the expression level of *pilA* gene was significantly decreased in the three *luxR* knockout mutants compared to wildtype, indicating that solo LuxRs can mediate Psa response to environmental AHL signaling molecules and regulate motility, biofilm formation and virulence (Cellini et al., 2019). In *L. brunescens*, the DSF quorum sensing system positively regulates the XSAC biosynthesis and surface motility, and the DSF quorum sensing system also positively regulates the expression of *peg.2863* and *pilA*_1_ (Ling et al., 2019). Taken together, the DSF quorum sensing system positively regulates the synthesis and motility of XSAC, while the AHL quorum sensing system affects cell growth and autolysis which further affecting the XSAC biosynthesis and surface motility.

## Conclusion

In this study, we report the role of the AHL quorum-sensing system in the regulation of cell growth and autolysis, and our data revealed the sucrose consumption and autolysis system to be negatively regulated by the AHL quorum-sensing system. First, we characterized three different AHLs in *L. brunescens*, including 3-OH-C_10_-HSL, C_8_-HSL and 3-oxo-C_6_-HSL. Second, we observed that the AHL mutants grew more rapidly than did wildtype OH23; the sucrose consumption rates were high, as was expression of the α-glucosidase gene in AHL mutants. These findings indicate that the AHL quorum-sensing system negatively regulates sucrose consumption and that the growth rate is increased in AHL QS mutants compared to wildtype OH23. Third, the autolysis rate of the AHL QS mutants was higher than that of wildtype, and expression of the key gene *peg.977* was increased in the AHL QS mutants, suggesting that the autolysis process was strongly influenced by the AHL quorum-sensing system. Moreover, XSAC production and surface motility might be affected by growth and autolysis and that the AHL quorum-sensing system indirectly regulates XSAC biosynthesis and surface motility. Altogether, the AHL quorum sensing system is involved in growth and autolysis regulation, indicating the AHL quorum sensing system regulates nutrition consumption to maintain population stable and cooperative in *L. brunescens*.

## Data Availability Statement

The datasets generated for this study can be found in the GenBank: MN181567, MN181568, MN557391, and MN557390.

## Author Contributions

JL, LZ, GW, YZ, and TJ conducted the experiments. FL designed and conducted the experiments and revised the manuscript. JL, LZ, GW, and YZ contributed to the writing of the manuscript.

## Conflict of Interest

The authors declare that the research was conducted in the absence of any commercial or financial relationships that could be construed as a potential conflict of interest.

## Footnotes

1 https://blast.ncbi.nlm.nih.gov/Blast.cgi

## Supplementary Material

FIGURE S1 ESI-MS/MS analysis of AHLs and AHL production in different time pints in *L. brunescens*. **(A)** According to the ESI-MS results, the structures of AHLs were calculated by using the following formulas: *n* = [(m/z-1-171)/28]C_*n ×2+4*_-HSL; *n* = [(m/z-1-187)/28] 3-hydroxyl-C_*n×2+4*_-HSL; *n* = [(m/z-1-185)/28] 3-oxo-C_*n×2+4*_-HSL (Bainton et al., 1992). **(B)** The supernatants of wildtype OH23 and its AHL mutants were collected at indicated time points, and AHL activities were measured by detection strain *A. tumefaciens* KYC55 (pJZ372) (pJZ384) (pJZ410).

FIGURE S2 Identification of genes involved in AHL quorum sensing system in *L. brunescens*. **(A)** Screening the genes related to AHL quorum sensing system and AHL productions of mutants were detected by using the detection strain *A. tumefaciens* KYC55 (pJZ372) (pJZ384) (pJZ410). ^∗∗∗^*P* < 0.001 (Student’s *t* test). **(B)** Results of Arbitrary PCR of mutants. **(C)** Identification of transposon insertion position and genetic map of *lbsR* and *lbsI*. The triangles represent the transposon insertion positions.

FIGURE S3 Sequence alignment and function identification of α-glucosidase in *L. brunescens*. **(A)** Amino acid sequence alignment of *peg.1602*. *L. brunescens*, MN557391; *Xanthomonadaceae* bacterium, TXH70081.1; *Lysobacter* sp. TY2-98, WP_115647852.1; *Thermomonas haemolytica*, WP_114958989.1. **(B)** SDS-PAGE of the different fractions in the purification of α-glucosidase from *L. brunescens*. **(C)** Enzyme activity detection of α-glucosidase from OH23.

FIGURE S4 Growth of *L. brunescens* AHL-complemented strains. **(A)** Growth of gentamicin-marked complemented strains. Gentamicin-marked wildtype OH23 [OH23 (pBBR)], AHL-complemented strains [Δ*lbsI* (*lbsI*) and Δ*lbsR* (*lbsR*)] and AHL control strains [Δ*lbsI* (pBBR) and Δ*lbsR* (pBBR)] (OD_600_ of approximately 1.0) were grown in NA liquid medium with gentamicin at 28°C, and the OD_600_ value was measured at the time points indicated. The data are the combination of three individual experiments. **(B)** Growth of chemical complemented strains. 2 μM (final concentration) C8-HSL was then added to the cultures of Δ*lbsI* treatments and the OD_600_ value was measured at the time points indicated. **(C)** α-glucosidase gene *peg.1602* expression in wildtype OH23, Δ*lbsI* and chemical complemented strain. The cells were collected at the OD_600_≈1.0 and RNA were extracted by using TRIzol solution (TaKaRa Biocompany). The real-time PCR assay was carried out by using QuantStudio 6 Flex Real-Time PCR System (Thermo Fisher Scientific) and HiScript II One Step qRT-PCR SYBR Green Kit (Vazyme Biotech Co., Ltd.). The data are the combination of three individual experiments. Statistical analyses were performed using Student’s *t*-test compared with wildtype OH23. ^∗∗∗^*P* < 0.001 and ns, *P* > 0.05 (Student’s *t*-test).

FIGURE S5 Peptidoglycan hydrolase gene *peg.977* expression in wildtype OH23, Δ*lbsI* and chemical complemented strain. The data are the combination of three individual experiments. Statistical analyses of *peg.1602* expression were performed using Student’s *t*-test compared with wildtype OH23. ^∗^*P* < 0.05 and ^∗∗^*P* < 0.01 (Student’s *t*-test).

FIGURE S6 Diameter of inhibition zone and surface motility ormalized by OD_600_. **(A)** Diameter of inhibition zone normalized by OD_600_. **(B)** Diameter of surface motility zone normalized by OD_600_. ^∗^*P* < 0.05; ^∗∗^*P* < 0.01; ^∗∗∗^*P* < 0.001; and ns, *P* > 0.05 (Student’s *t*-test).
