# Supplementary figures and images for: The AHL Quorum-Sensing System Negatively Regulates Growth and Autolysis in Lysobacter brunescens
Source: Front Microbiol. 2019 Dec 3;10:2748. doi: 10.3389/fmicb.2019.02748 (PMC6902743; doi:10.3389/fmicb.2019.02748)

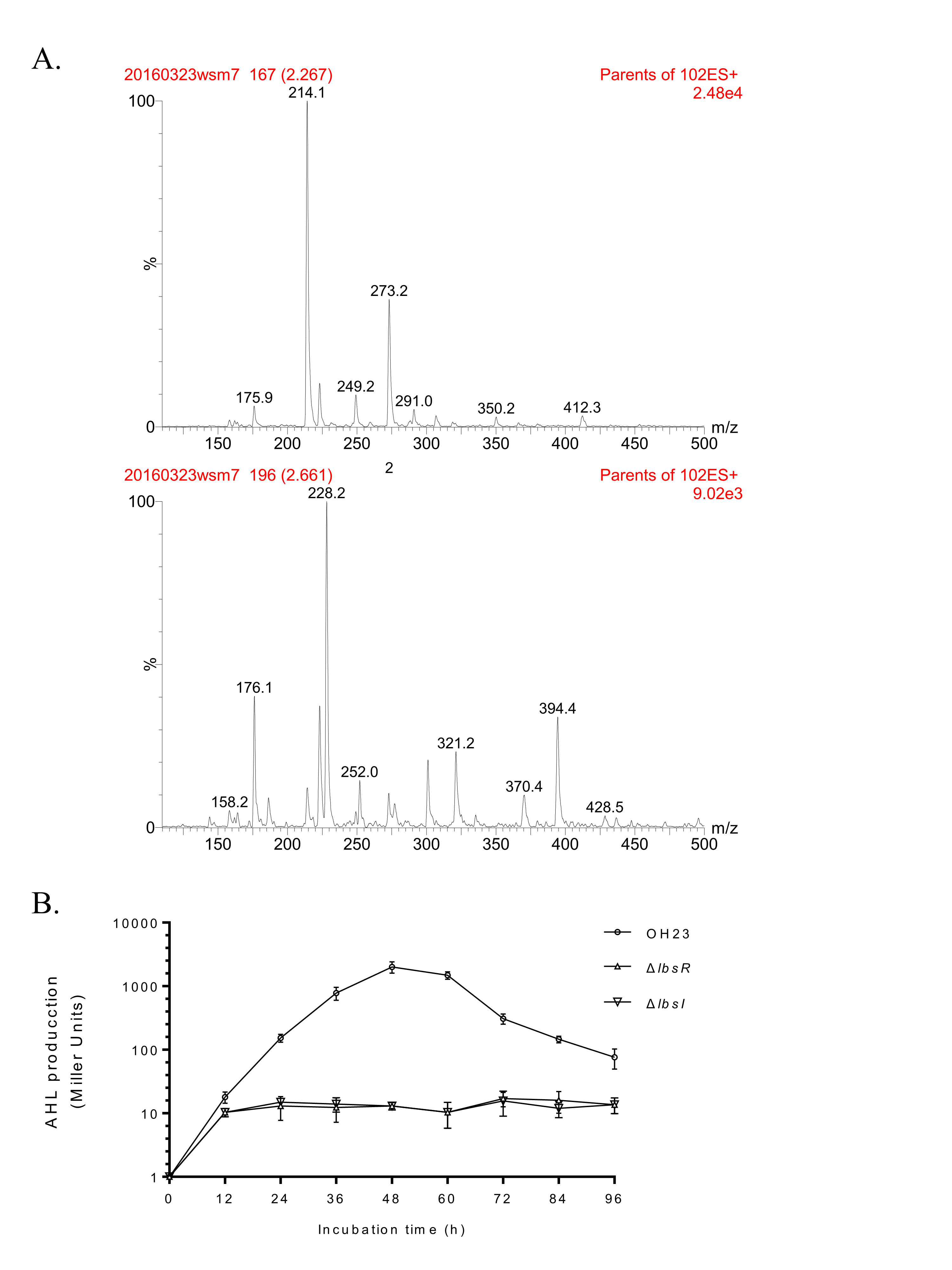

Supplement: FIGURE S1 — ESI-MS/MS analysis of AHLs and AHL production in different time pints in L. brunescens. (A) According to the ESI-MS results, the structures of AHLs were calculated by using the following formulas: n = [(m/z-1-171)/28]Cn ×2+4-HSL; n = [(m/z-1-187)/28] 3-hydroxyl-Cn×2+4-HSL; n = [(m/z-1-185)/28] 3-oxo-Cn×2+4-HSL (Bainton et al., 1992). (B) The supernatants of wildtype OH23 and its AHL mutants were collected at indicated time points, and AHL activities were measured by detection strain A. tumefaciens KYC55 (pJZ372) (pJZ384) (pJZ410). [file Image_1.TIF]

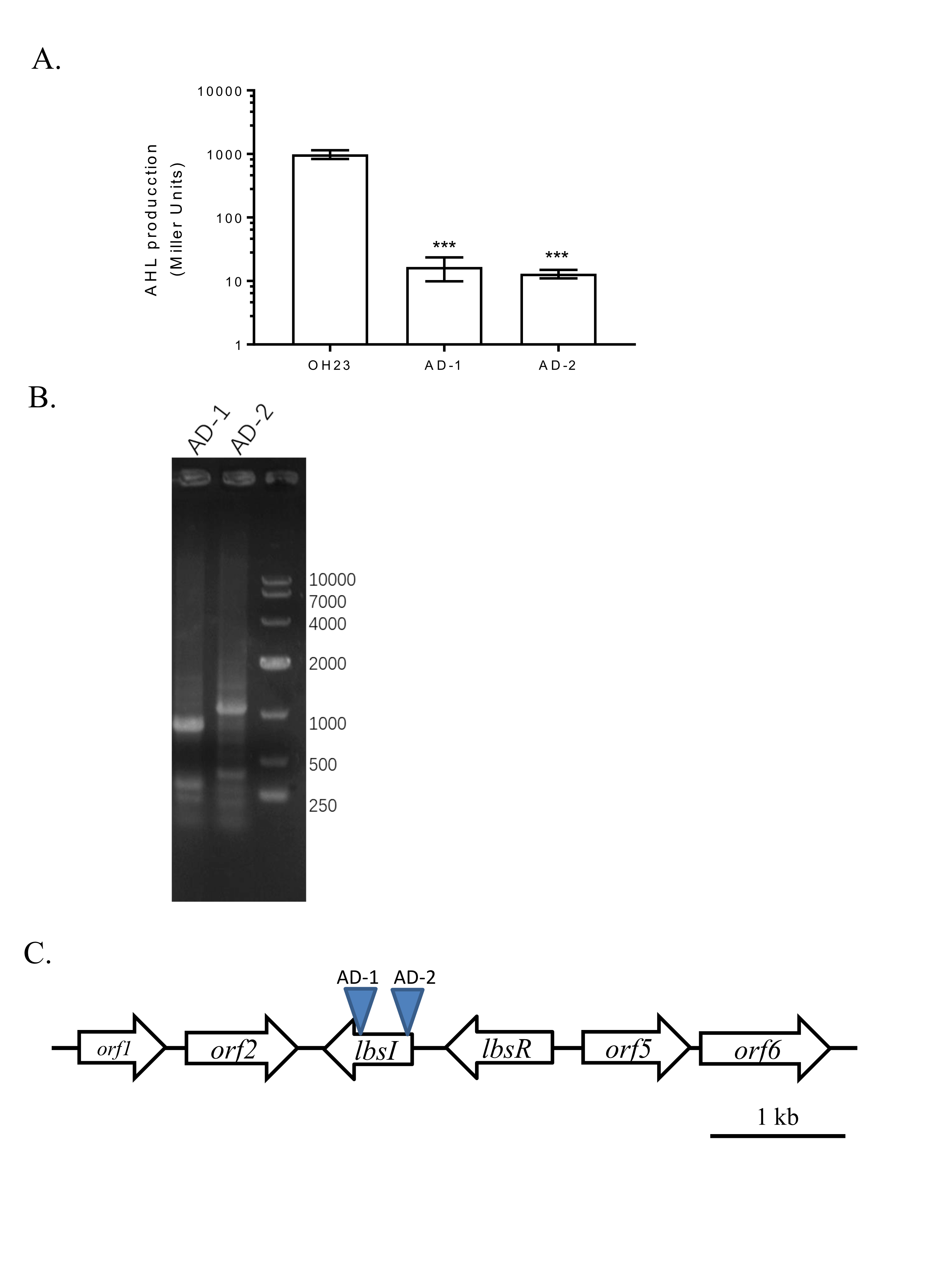

Supplement: FIGURE S2 — Identification of genes involved in AHL quorum sensing system in L. brunescens. (A) Screening the genes related to AHL quorum sensing system and AHL productions of mutants were detected by using the detection strain A. tumefaciens KYC55 (pJZ372) (pJZ384) (pJZ410). ∗∗∗P < 0.001 (Student’s t test). (B) Results of Arbitrary PCR of mutants. (C) Identification of transposon insertion position and genetic map of lbsR and lbsI. The triangles represent the transposon insertion positions. [file Image_2.TIF]

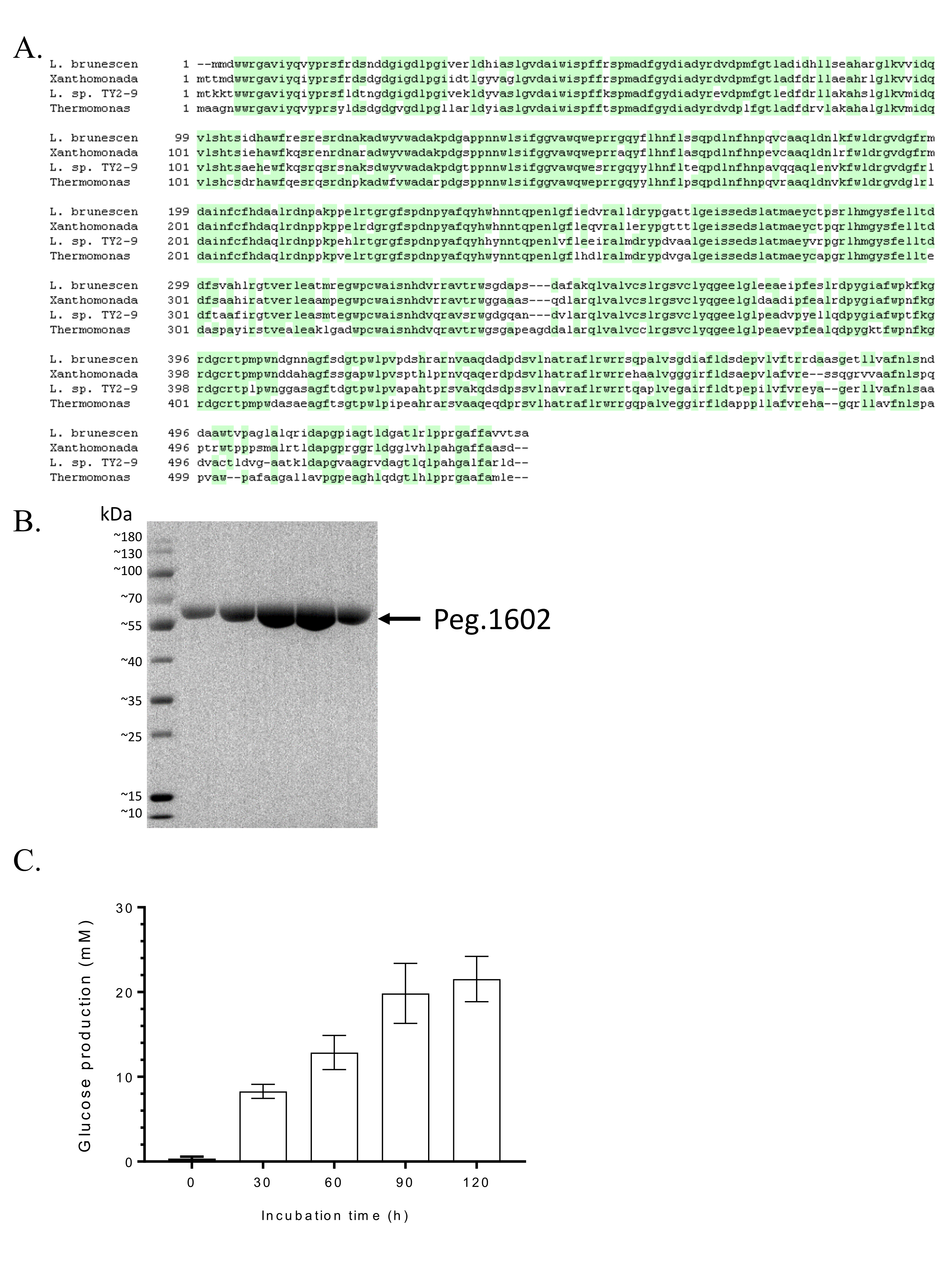

Supplement: FIGURE S3 — Sequence alignment and function identification of α-glucosidase in L. brunescens. (A) Amino acid sequence alignment of peg.1602. L. brunescens, MN557391; Xanthomonadaceae bacterium, TXH70081.1; Lysobacter sp. TY2-98, WP_115647852.1; Thermomonas haemolytica, WP_114958989.1. (B) SDS-PAGE of the different fractions in the purification of α-glucosidase from L. brunescens. (C) Enzyme activity detection of α-glucosidase from OH23. [file Image_3.TIF]

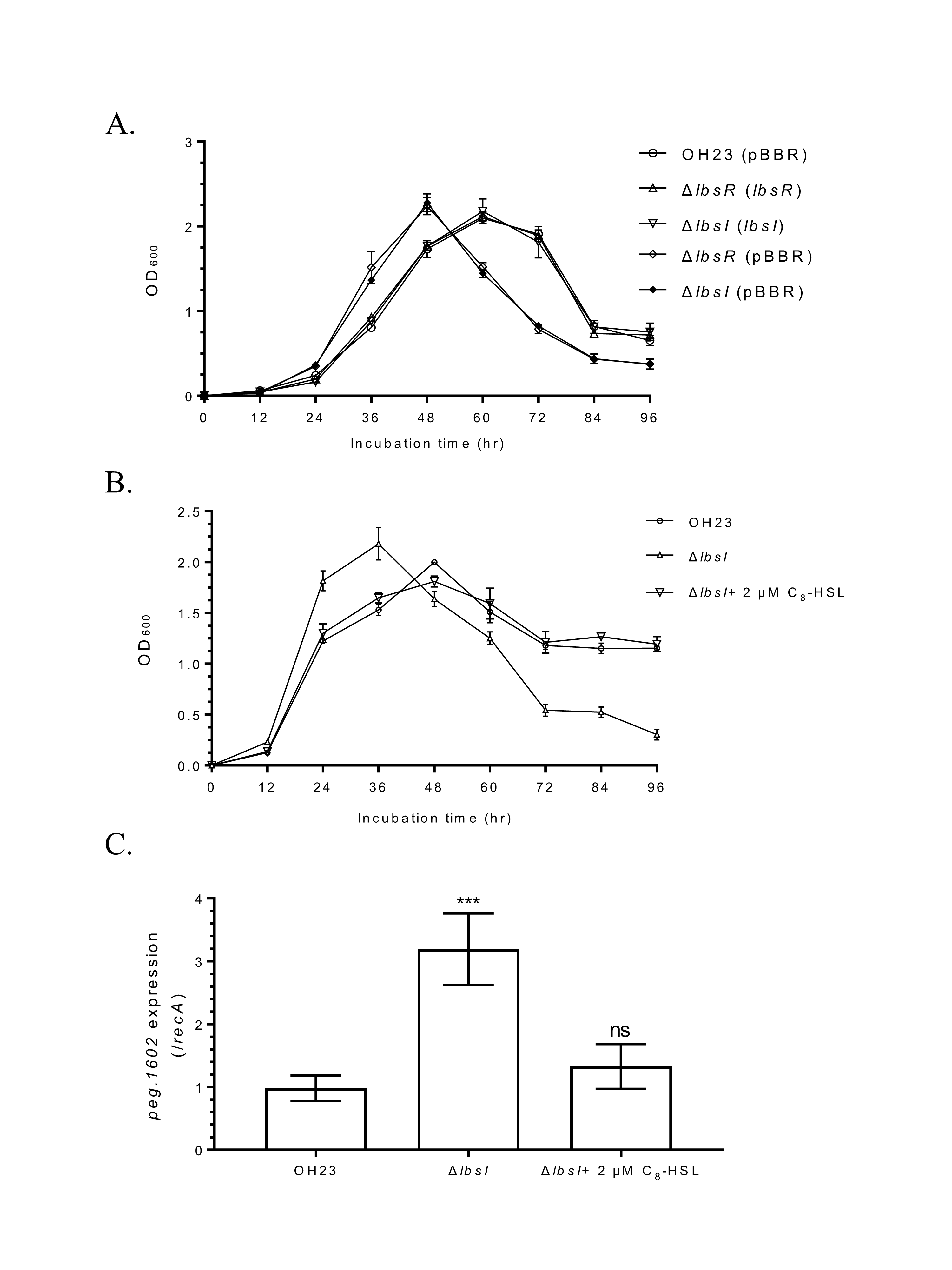

Supplement: FIGURE S4 — Growth of L. brunescens AHL-complemented strains. (A) Growth of gentamicin-marked complemented strains. Gentamicin-marked wildtype OH23 [OH23 (pBBR)], AHL-complemented strains [ΔlbsI (lbsI) and ΔlbsR (lbsR)] and AHL control strains [ΔlbsI (pBBR) and ΔlbsR (pBBR)] (OD600 of approximately 1.0) were grown in NA liquid medium with gentamicin at 28°C, and the OD600 value was measured at the time points indicated. The data are the combination of three individual experiments. (B) Growth of chemical complemented strains. 2 μM (final concentration) C8-HSL was then added to the cultures of ΔlbsI treatments and the OD600 value was measured at the time points indicated. (C) α-glucosidase gene peg.1602 expression in wildtype OH23, ΔlbsI and chemical complemented strain. The cells were collected at the OD600≈1.0 and RNA were extracted by using TRIzol solution (TaKaRa Biocompany). The real-time PCR assay was carried out by using QuantStudio 6 Flex Real-Time PCR System (Thermo Fisher Scientific) and HiScript II One Step qRT-PCR SYBR Green Kit (Vazyme Biotech Co., Ltd.). The data are the combination of three individual experiments. Statistical analyses were performed using Student’s t-test compared with wildtype OH23. ∗∗∗P < 0.001 and ns, P > 0.05 (Student’s t-test). [file Image_4.TIF]

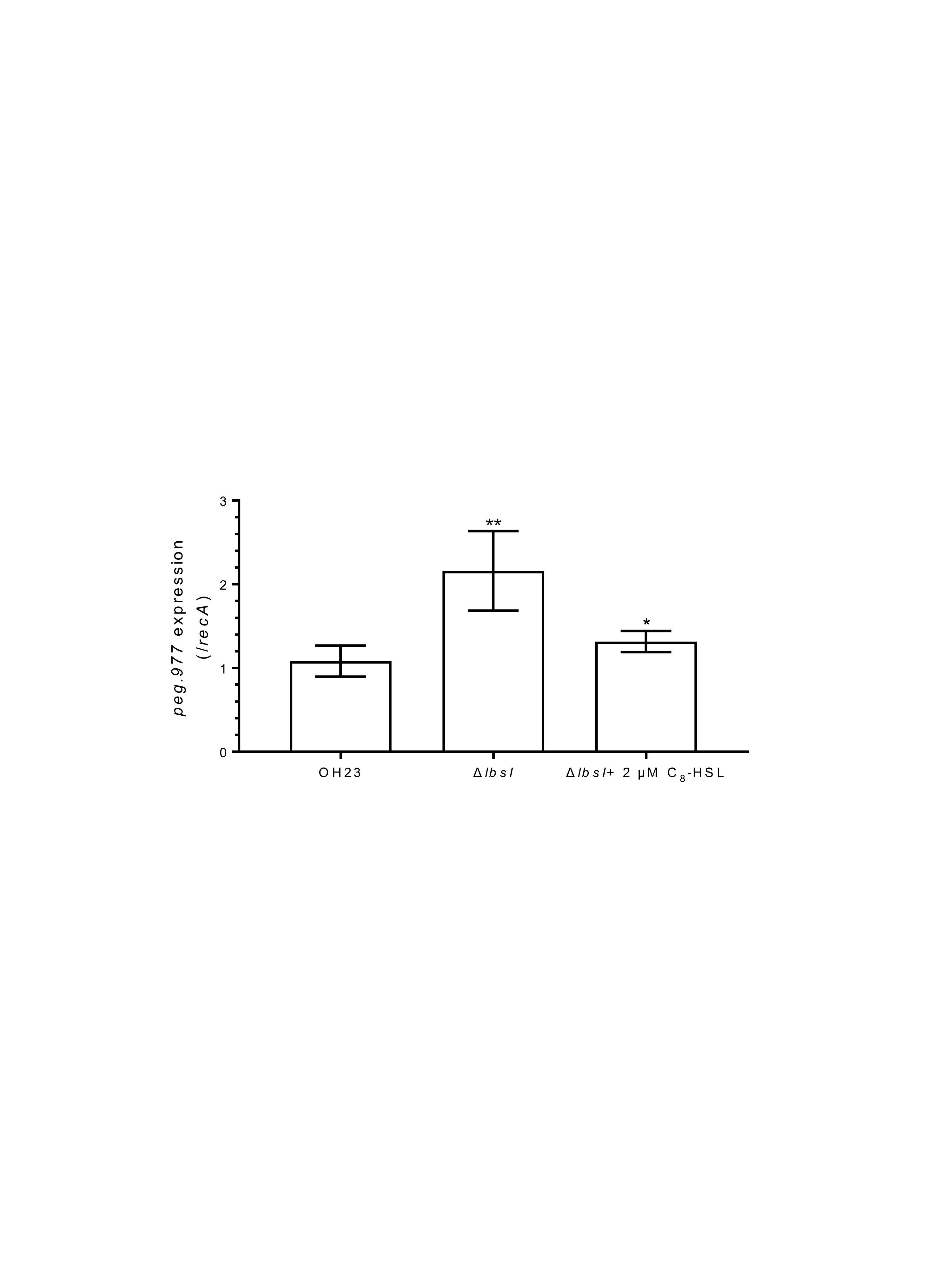

Supplement: FIGURE S5 — Peptidoglycan hydrolase gene peg.977 expression in wildtype OH23, ΔlbsI and chemical complemented strain. The data are the combination of three individual experiments. Statistical analyses of peg.1602 expression were performed using Student’s t-test compared with wildtype OH23. ∗P < 0.05 and ∗∗P < 0.01 (Student’s t-test). [file Image_5.TIF]

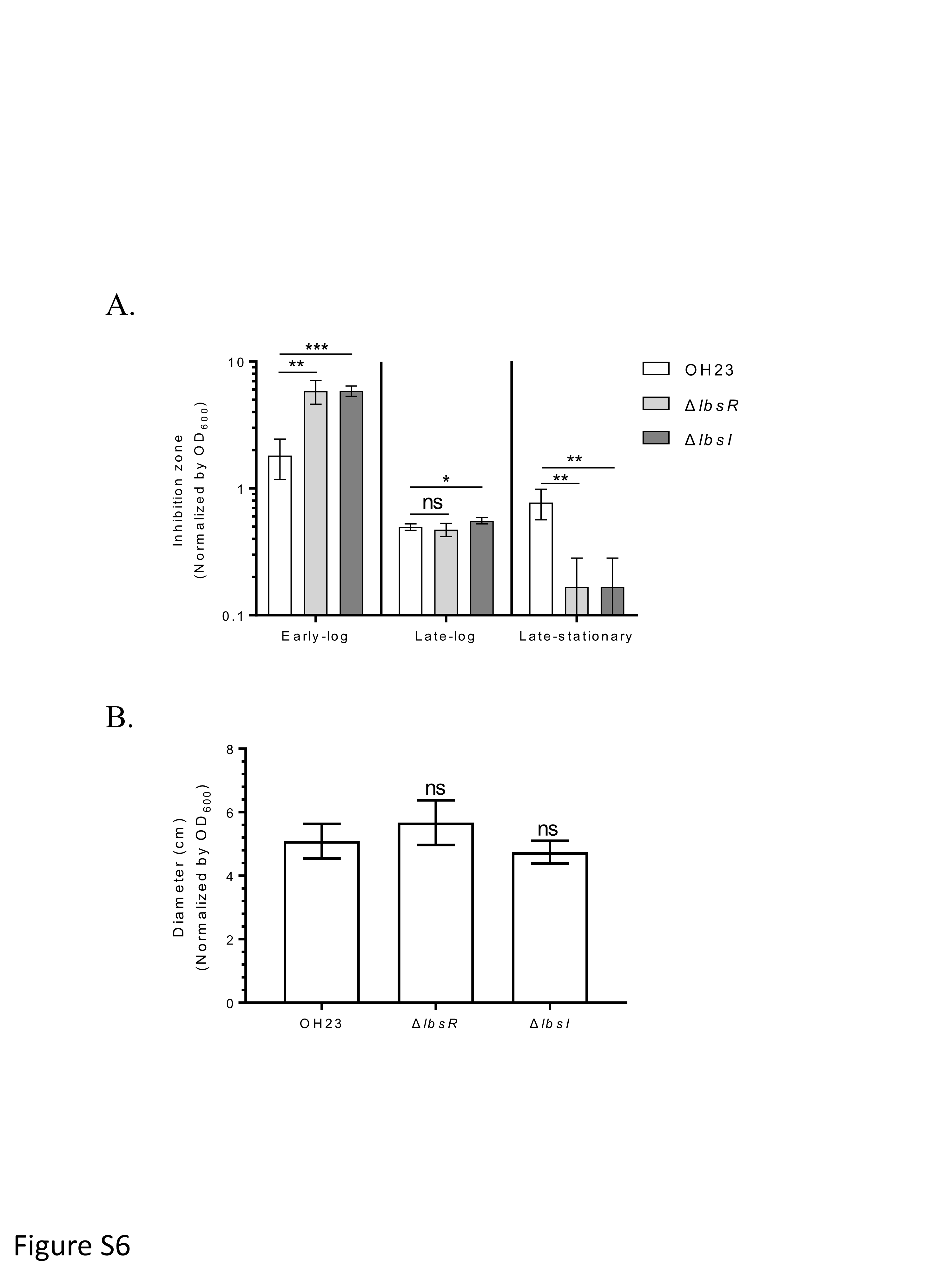

Supplement: FIGURE S6 — Diameter of inhibition zone and surface motility ormalized by OD600. (A) Diameter of inhibition zone normalized by OD600. (B) Diameter of surface motility zone normalized by OD600. ∗P < 0.05; ∗∗P < 0.01; ∗∗∗P < 0.001; and ns, P > 0.05 (Student’s t-test). [file Image_6.TIF]
