# Supplementary material for: The AHL Quorum-Sensing System Negatively Regulates Growth and Autolysis in Lysobacter brunescens
Source: Front Microbiol. 2019 Dec 3;10:2748. doi: 10.3389/fmicb.2019.02748 (PMC6902743; doi:10.3389/fmicb.2019.02748)
Supplement: Supplementary file 7 [file Table_1.docx]

**The AHL Quorum-sensing System Negatively Regulates Growth and Autolysis** **in *Lysobacter brunescens***

Jun Ling^a,#^, Lan Zhou^b,#^, Guichun Wu^a^, Yancun Zhao^a^, Tianping Jiang^a^, Fengquan Liu^a,c,*^

^a^ Institute of Plant Protection, Jiangsu Academy of Agricultural Sciences, Jiangsu Key Laboratory for Food Quality and Safety-State Key Laboratory Cultivation Base of Ministry of Science and Technology, Nanjing 210014, People’s Republic of China

^b^ Academy of Agricultural Sciences of Yanbian, Longjing Jilin 133400, People’s Republic of China

^c^ School of Environment and Safety Engineering, Jiangsu University, Zhenjiang 212013, People’s Republic of China

^#^ Both authors contributed equally to this work.

^*^ To whom correspondence should be addressed. E-mail: [fqliu20011@sina.com](mailto:fqliu20011@sina.com)

**Supplemental Methods and Materials**

**AHL-deficient mutant screening**

Random transposon insertional libraries were constructed as described previously, with modifications (Judson and Mekalanos, 2000). Briefly, the SM10λpir strain containing pSC137, which carries a constitutively expressed chloramphenicol resistance gene, was used as the donor, and the rifamycin-resistant strain OH23 was used as the recipient. The transconjugants were selected on NB solid plates containing rifamycin and chloramphenicol. Random mutants were grown in liquid NB medium until the OD_600_ was approximately 1.0 and assayed using the liquid AHL bioassays mentioned above. The transposon insertion site-screened AHL-deficiency mutant was generated as described previously (Cao et al., 2009). Briefly, genomic DNA of the mutant was digested using *EcoR*I, ligated to the *EcoR*I-digested vector pBluescript (Clontech) and selected on LB solid plates containing ampicillin and chloramphenicol. The vector was sequenced by using the primer walking method, and the sequences were compared in the GenBank DNA sequence database.

**Growth measurements of complemented strains**

Because the addition of gentamicin affected the growth rate of test strains, OH23 (pBBR) (OH23 carrying the empty pBBR1-MCS5 vector) was used as a control to detect the growth rate of complementation strains. All the *L. brunescens* strains were cultured in NB medium at 28°C with shaking at 180 rpm until the OD_600_ was approximately 1.0 (which corresponds to approximately 10^9^ CFU/ml. One milliliter of culture for each strain was transferred to 50 ml of new liquid NB medium contained 8 μg/ml gentamicin, and the cultures were incubated at 28°C with shaking at 180 rpm. For the C8-HSL added treatments, the C8-HSL was added to the liquid NB medium to a final concentration at 2 μM. To measure growth, the OD_600_ value was determined every 12 h for each culture using a BioPhotometer Plus (Eppendorf, Germany) until each culture reached the stationary phase. Three replicates were performed for each treatment, and the experiment was repeated three times.

**Cloning and expression α-glucosidase (Peg.1602).**

The fragment of *peg.1602* was amplified by PCR using the following primers: 5’-CATCATCATCATCATCATATGGACTGGTGGCGCGGCGCGGTGATC-3’ and 5’-AGCTTGAATTCGGATCCTCAGGCGCTGGTGACCACCG-3’, and ligated into pCOLD II (Takara). The recombinant plasmid was transferred into *E.coli* BL21 (DE3) by electroporation for constructing the overexpression strain. The BL21 (DE3) containing the pCOLD-*peg.1602* were grown in liquid LB medium at 37ºC to OD_600_ ≈ 0.5. 0.5 mM (final concentration) IPTG was then added to the cultures and incubated at 15ºC for 24 h. The cells were collected by centrifugation at 4ºC and cell lysis was carried out by using an Ultrasonic Disruptor SCIENTZ-IID (Scientz, China). The target protein was purified by TALON metal affinity column (Takara) and eluted with elution buffer containing 300 mM imidazole. Proteins were dialyzed to remove the imidazole and stored at -20ºC for further use. The concentration of Peg.1602 protein was calculated using a BCA Protein Assay kit (Takara).

**Activity detection of α-glucosidase (Peg.1602).**

Sucrose was added to Tris-NaCl buffer to a final concentration at 50 mM and 5 μl of purified Peg.1602 protein (2.21 μg/μl) was added to the Tris-NaCl buffer and incubated at 28ºC. The samples were collected at indicated time points and the concentration of glucose was detected by using a glucose detection kit (Solarbio, Beijing Solarbio Science &Technology Co., Ltd.). The concentration of glucose was represented the activity of α-glucosidase (Peg.1602).

**Reference**

Cao, H., Yang, M., Zheng, H., Zhang, J., Zhong, Z., and Zhu, J. (2009). Complex quorum-sensing regulatory systems regulate bacterial growth and symbiotic nodulation in Mesorhizobium tianshanense. *Arch Microbiol* 191**,** 283-289.

Judson, N., and Mekalanos, J.J. (2000). Transposon-based approaches to identify essential bacterial genes. *Trends Microbiol* 8**,** 521-526.
